# Identification of CNGB1 as a Predictor of Response to Neoadjuvant Chemotherapy in Muscle-Invasive Bladder Cancer

**DOI:** 10.3390/cancers13153903

**Published:** 2021-08-02

**Authors:** Anastasia C. Hepburn, Nicola Lazzarini, Rajan Veeratterapillay, Laura Wilson, Jaume Bacardit, Rakesh Heer

**Affiliations:** 1 Translational and Clinical Research Institute, Newcastle University Centre for Cancer, Newcastle University, Newcastle upon Tyne NE2 4HH, UK; anastasia.hepburn@ncl.ac.uk (A.C.H.); laura.wilson5@newcastle.ac.uk (L.W.); 2 ICOS Research Group, School of Computing, Newcastle University, Newcastle upon Tyne NE4 5TG, UK; nicola.lazzarini@iqvia.com (N.L.); jaume.bacardit@newcastle.ac.uk (J.B.); 3 Department of Urology, Freeman Hospital, The Newcastle upon Tyne Hospitals NHS Foundation Trust, Newcastle upon Tyne NE7 7DN, UK; veeratterapillayr@doctors.org.uk

**Keywords:** muscle invasive bladder cancer, neoadjuvant chemotherapy, cisplatin, biomarker, machine learning, CNGB1

## Abstract

**Simple Summary:**

Chemotherapy is recommended prior to surgical removal of the bladder for muscle-invasive bladder cancer patients. Despite a survival benefit, some patients do not respond and experience substantial toxicity and delay in surgery. Therefore, the identification of chemotherapy responders before initiating therapy would be a helpful clinical asset. To date, there are no reliable biomarkers routinely used in clinical practice that identify patients most likely to benefit from chemotherapy and their identification is urgently required for more precise delivery of care. To address this issue, we compared gene expression profiles of biopsy materials from 30 chemotherapy-responder and -non-responder patients. This analysis revealed a novel signature gene set and CNGB1 as a simpler proxy as a promising biomarker to predict chemoresponsiveness of muscle-invasive bladder cancer patients. Our findings require further validation in larger patient cohorts and in a clinical trial setting.

**Abstract:**

Cisplatin-based neoadjuvant chemotherapy (NAC) is recommended prior to radical cystectomy for muscle-invasive bladder cancer (MIBC) patients. Despite a 5–10% survival benefit, some patients do not respond and experience substantial toxicity and delay in surgery. To date, there are no clinically approved biomarkers predictive of response to NAC and their identification is urgently required for more precise delivery of care. To address this issue, a multi-methods analysis approach of machine learning and differential gene expression analysis was undertaken on a cohort of 30 MIBC cases highly selected for an exquisitely strong response to NAC or marked resistance and/or progression (discovery cohort). RGIFE (ranked guided iterative feature elimination) machine learning algorithm, previously demonstrated to have the ability to select biomarkers with high predictive power, identified a 9-gene signature (*CNGB1*, *GGH*, *HIST1H4F*, *IDO1*, *KIF5A*, *MRPL4*, *NCDN*, *PRRT3*, *SLC35B3*) able to select responders from non-responders with 100% predictive accuracy. This novel signature correlated with overall survival in meta-analysis performed using published NAC treated-MIBC microarray data (validation cohort 1, *n* = 26, Log rank test, *p* = 0.02). Corroboration with differential gene expression analysis revealed cyclic nucleotide-gated channel, *CNGB1*, as the top ranked upregulated gene in non-responders to NAC. A higher CNGB1 immunostaining score was seen in non-responders in tissue microarray analysis of the discovery cohort (*n* = 30, *p* = 0.02). Kaplan-Meier analysis of a further cohort of MIBC patients (validation cohort 2, *n* = 99) demonstrated that a high level of CNGB1 expression associated with shorter cancer specific survival (*p* < 0.001). Finally, in vitro studies showed siRNA-mediated CNGB1 knockdown enhanced cisplatin sensitivity of MIBC cell lines, J82 and 253JB-V. Overall, these data reveal a novel signature gene set and *CNGB1* as a simpler proxy as a promising biomarker to predict chemoresponsiveness of MIBC patients.

## 1. Introduction

Bladder cancer is the tenth commonest cancer worldwide with 549,000 new cases and 199,000 deaths reported in 2018 [1]. While 85% of patients present with less aggressive non-muscle invasive bladder cancer (NMIBC), they have a high risk of recurrence (50–70%) and up to 25% will progress to more advanced disease [2]. For patients that present with, or progress to, muscle invasive bladder cancer (MIBC), the mainstay of treatment is radical cystectomy and radiotherapy [3]. However, 5-year disease-free survival is as low as 15–35% and up to 50% of patients develop metastasis within two years of surgery inevitably succumbing to their disease [4]. Failure is usually due to occult micrometastatic disease present at diagnosis. Cisplatin-based neoadjuvant chemotherapy (NAC), including administering regimens such as MVAC (Methotrexate, Vinblastine, Doxorubicin and Cisplatin), is a promising strategy to achieve pathological downstaging as well as early eradication of micrometastasis to improve patient survival [3].

High level evidence from two large, randomised trials and two meta-analyses demonstrated that the MVAC regimen prior to cystectomy resulted in a 5–10% increase in 5-year cancer-specific survival (CSS) in comparison to cystectomy alone [5,6,7,8]. Interestingly, the 5-year CSS for responders to NAC is 90% in contrast to 30–40% for non-responders. However, only approximately 40% of patients will have a major response to NAC (defined as absence of muscle-invasive disease and lymph node metastasis; <pT2 and pN0) and benefit from it. Furthermore, non-responders suffer substantial overtreatment, delay of surgery and loss of opportunity for further therapy due to physical deterioration from toxicity or to disease progression. Therefore, identification of a reliable method to stratify NAC administration based on predicted response is of critical importance for the management of MIBC patients and may ultimately lead to future personalised medicine. Recent studies have explored DNA repair gene mutations (*ERCC1*, *ERCC2*, *BRCA1*) [9,10,11,12], regulators of apoptosis (survivin, Bcl-xL) [13], receptor tyrosine kinase mutations (*ERBB2*) [14], gene expression signatures [13,15,16,17,18,19], molecular subtypes of bladder cancer [20,21,22,23] and alterations in the cellular mechanisms of drug uptake/transport (CTR-1, MDR1) [24,25] as potential predictors of response to NAC and offer promise for improving patient selection for such treatment and clinical outcomes. However, to date, no biomarker exists in the clinical setting to prospectively identify the patients most likely to benefit from NAC.

**Figure 1:**
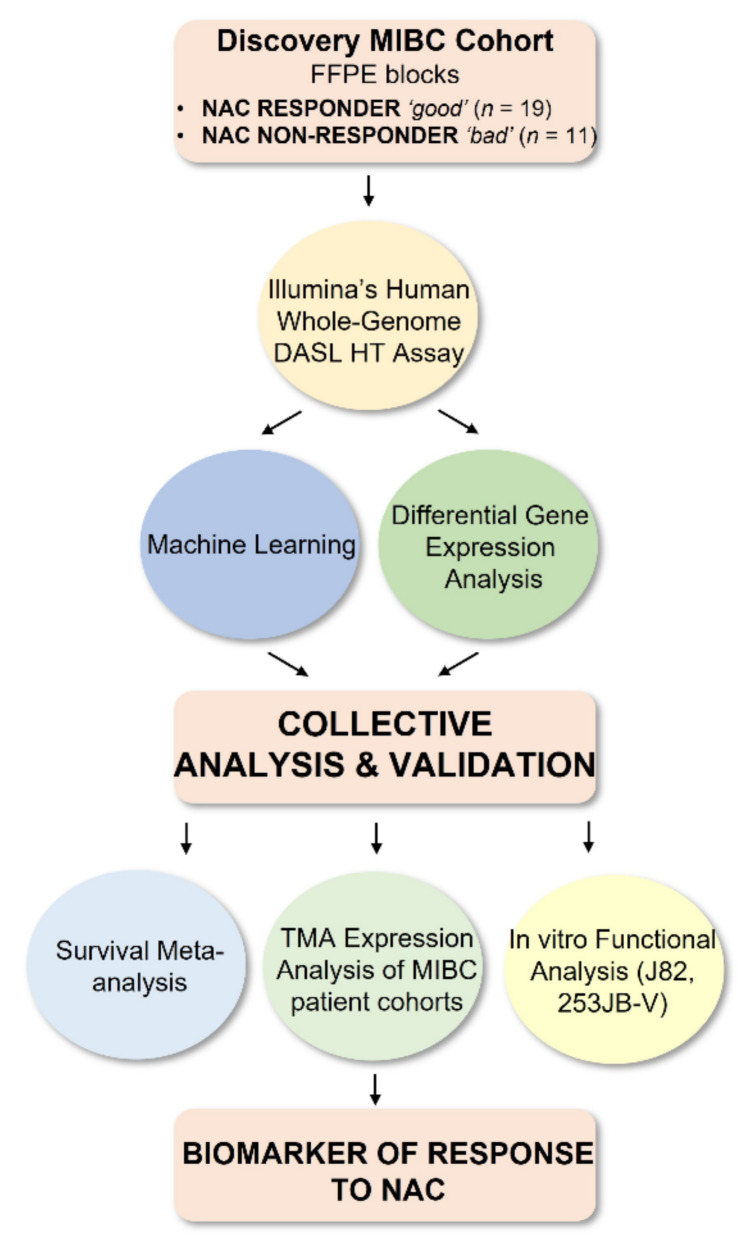
Overview of the study. This study represents a multi-methods analysis approach of machine learning and differential gene expression analysis following performance of gene expression profiling on a cohort of MIBC patients highly selected for an exquisitely strong response to NAC (responder) or marked resistance and/or progression (non-responder). Identified gene signatures and top ranked gene markers were subjected to validation using meta-analysis, immunohistochemistry and in vitro functional assays. FFPE, formalin-fixed paraffin-embedded; TMA, tissue microarray analysis; MIBC, muscle-invasive bladder cancer; NAC, neoadjuvant chemotherapy.

In this study, we aimed to identify a biomarker to predict response to NAC in MIBC patients. Biomarker discovery investigations using omics technologies such as microarrays, next generation sequencing and mass spectroscopy generate large volumes of data for analysis with bioinformatic tools. A wide range of bioinformatics approaches have flourished, including machine learning methods which are concerned with the development and application of computer algorithms whose prediction performance improves with experience [26]. Machine learning methods, and particularly feature selection algorithms, have been extensively used in biomarker discovery and demonstrated to effectively identify small but relevant subsets of variables from transcriptomic data sets [27,28,29]. Specifically in this study, RGIFE (ranked guided iterative feature elimination) machine learning algorithm [27] was applied to microarray gene expression data from 30 MIBC cases who received NAC and identified a 9-gene signature (*CNGB1*, *GGH*, *HIST1H4F*, *IDO1*, *KIF5A*, *MRPL4*, *NCDN*, *PRRT3*, *SLC35B3*) able to differentiate responders from non-responders with 100% predictive accuracy. This signature also associated with outcome in meta-analysis performed using published microarray data. Next, a complementary approach of traditional gene ranking by differential gene expression analysis revealed *CNGB1*, encoding a cyclic nucleotide-gated channel that regulates intracellular cation (Ca^2+^) flow and downstream transduction signalling cascades, as the top ranked upregulated gene in non-responders to NAC and its expression was validated in a further additional cohort of MIBC patients and in in vitro studies of MIBC cancer cell lines. In Figure 1, we provide an overview of the analyses conducted in this study.

## 2. Results

### 2.1. Machine Learning Approach to Identify Gene Signatures Predicting Response to NAC

**Figure 2:**
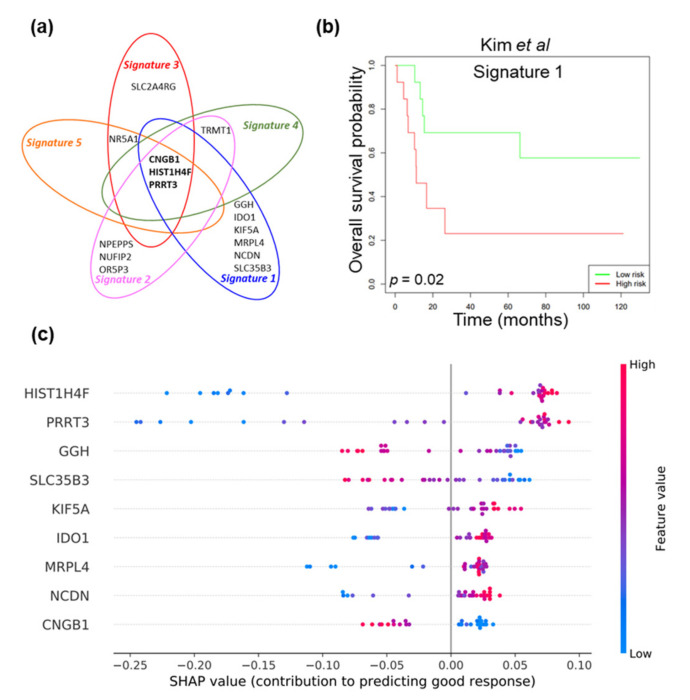
Machine learning approach to identify gene signatures predicting response to NAC. (**a**) Gene commonality of signatures identified by RGIFE machine learning algorithm, (**b**) A Cox proportional hazards regression analysis was performed to assess the relationship between expression of Signature 1 and overall survival in the Kim et al. dataset (Validation cohort 1) [30]. The model generated a survival risk estimate from the panel of markers comprising Signature 1 based on survival time and overall survival status. The model assigned a survival risk to each sample. The cohort was divided into two equally sized groups by the median risk (‘low risk’ and ‘high risk’). Kaplan-Meier plot shows Signature 1 significantly associated with overall survival in MIBC patients treated with NAC (*n* = 26, Log rank test, *p* = 0.02) in Validation cohort 1, (**c**) SHAP summary plot showing the contribution that each gene of Signature 1 has in the prediction of good response for each patient sample in the Discovery cohort (SHAP value, the estimation of the contribution that each specific gene has in the prediction of good response for a given sample). A positive SHAP value indicates contribution to predicting good response to NAC. A negative SHAP value reflects a lack of support for predicting good response to NAC. i.e., contributes to predicting bad response. Blue and red points indicate low and high expressions of a gene, respectively.

Gene expression profiling using Illumina’s Human Whole-Genome DASL HT Assay was carried out on 30 MIBC patients that received NAC (Discovery cohort). Clinicopathological characteristics are summarised in Table 1. These patients had large burden of disease with documented incomplete resection specifically selected for an exquisitely strong chemotherapeutic response or marked resistance and/or progression, therefore providing a relevant cohort to investigate neoadjuvant administration of chemotherapy in improving MIBC patient survival. Based on their response to treatment, patients were categorised into two groups: ‘responders’, patients who achieved downstaging (≤pT1) and ‘non-responders’, patients who did not achieve downstaging (≥pT2). Microarray gene expression profiles of tumours from 19 ‘responders’ and 11 ‘non-responders’ were compared. The RGIFE machine learning algorithm was applied to the microarray dataset with the aim to identify new biomarkers to predict response to NAC (see Materials and Methods Section 4.3 for detailed description of the algorithm). RGIFE has been demonstrated to have the capacity to select few biomarkers with high predictive power by utilising an iterative process that discards features if their removal does not decrease the overall predictive performance of the computational model [27]. Accordingly, RGIFE identified five gene signatures able to classify the two patient groups with prediction accuracies of 0.933 to 1.000 (Table 2). *CNGB1* (cyclic nucleotide gated channel beta 1), *HIST1H4F* (histone cluster 1, H4 family member F) and *PRRT3* (proline-rich transmembrane protein 3) were genes shared by all signatures (Figure 2a). Next, signature evaluation was undertaken in published bladder cancer gene expression dataset by Kim and colleagues comprising 62 MIBC patients with available survival information (GSE13507, Validation cohort 1, Figure 2b) [30]. Signature 1 (*CNGB1*, *GGH*, *HIST1H4F*, *IDO1*, *KIF5A*, *MRPL4*, *NCDN*, *PRRT3*, *SLC35B3*) significantly associated with overall survival in MIBC patients treated with NAC (*n* = 26, Log rank test, *p* = 0.02) as demonstrated by Kaplan Meier analysis. Additionally, a SHAP (SHapley Additive exPlanations) summary plot for Signature 1 provided further insight on the contribution of each gene to response to NAC for patients in the discovery cohort (Figure 2c). Briefly, such plots are informative in identifying if high/low expression of a particular gene is strongly associated with a positive or negative outcome in the prediction model (see Materials and Methods Section 4.5 for detailed description of SHAP). Particularly, high level expression of *HIST1H4F* and *PRRT3* indicated prediction of a ‘good’ response to NAC, whereas high level expression of *CNGB1* indicated prediction of ‘bad’ response to NAC. In summary, this machine learning approach identified a signature able to classify patients based on response to NAC that also associated with patient survival and moreover identified novel potential biomarkers of NAC response for MIBC patients.

### 2.2. Differential Gene Expression Analysis to Corroborate Machine Learning Data

**Figure 3:**
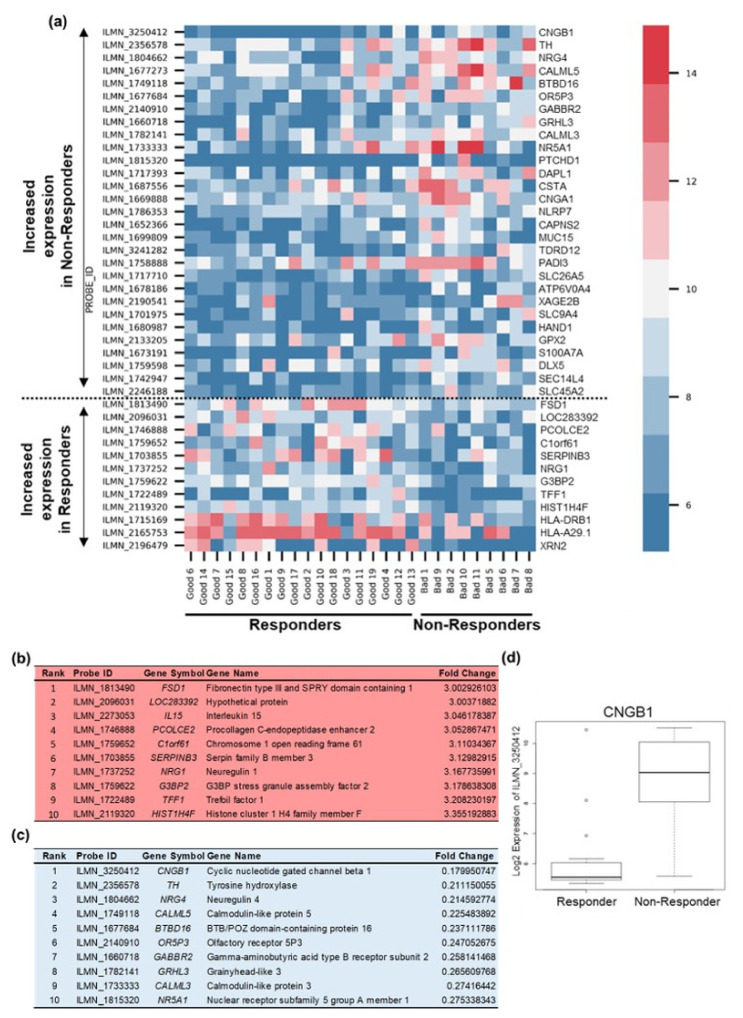
Differential gene expression analysis to corroborate machine learning data. (**a**) Heat map of genes differentially expressed by ‘responders’ and ‘non-responders’ in the Discovery cohort. Rows, single gene; columns, single patient. Each cell in the matrix represents the expression level of a single transcript in a single sample with red indicating upregulation and blue indicating downregulation compared with the median expression for that gene across all samples. (**b**) Top 10 genes upregulated (>3-fold) in ‘Responders’ to NAC. For full list, see Table S1. (**c**) Top 10 genes upregulated in ‘Non-Responders’ to NAC. For full list, see Table S2. (**d**) Box plot demonstrating *CNGB1* gene expression was upregulated in ‘Non-Responders’ compared to ‘Responders’ (*p* = 0.029).

To contrast and further validate the findings from the machine learning approach, we next undertook a classical and non-machine learning approach of conventional differential gene expression analysis of the Discovery cohort. This approach enabled us to capitalise on the strengths of each method and aid in the more accurate evaluation of predictive biomarker candidates. Gene ranking identified genes differentially expressed between ‘responders’ and non-responders’ to NAC (Figure 3a). Ranked gene lists were generated and revealed *FSD1* (fibronectin type III and SPRY domain containing 1) as the top ranked upregulated gene in ‘responders’ and *CNGB1* as the top ranked upregulated gene in ‘non-responders’ to NAC (Figure 3b–d, Tables S1 and S2). *CNGB1* was shared by all five signatures identified by RGIFE (Figure 2a and Table 2) and its high expression indicated as a predictor of ‘bad’ response to NAC by SHAP analysis (Figure 2c). Additionally, two further genes ranked in the list of genes upregulated in ‘non-responders’ were also identified in three of the signatures selected by RGIFE (Figure 3c and Table 2). Specifically, *OR5P3* (olfactory receptor 5P3) was identified in signature 2 and *NR5A1* (nuclear receptor subfamily 5, group A, member 1) in signatures 3 and 5. Other genes upregulated in ‘non-responders’ included a further cyclic nucleotide gated channel and interacting partner of *CNGB1*, *CNGA1*, receptor tyrosine kinase ligand *NRG4*, calcium-sensing *CALML3*, *CALML5* and *S100A7A*, apoptosis associated *DAPL1* and solute transporters *SLC26A5*, *SLC9A4* and *SLC45A2* (Figure 3c, Table S2). Of note, *HIST1H4F*, identified by RGIFE in all five signatures and as a predictor of response by SHAP analysis, was upregulated in ‘responders’ (Figure 2a,c and Figure 3b and Table 2). Other genes upregulated in ‘responders’ included receptor tyrosine kinase ligand *NRG1*, protease inhibitor *SERPINB3*, trefoil factor *TFF1* and exoribonuclease *XRN2* (Figure 3b, Table S1).

### 2.3. CNGB1 Is Upregulated in ‘Non-Responders’ to NAC and Associates with MIBC Patient Survival

**Figure 4:**
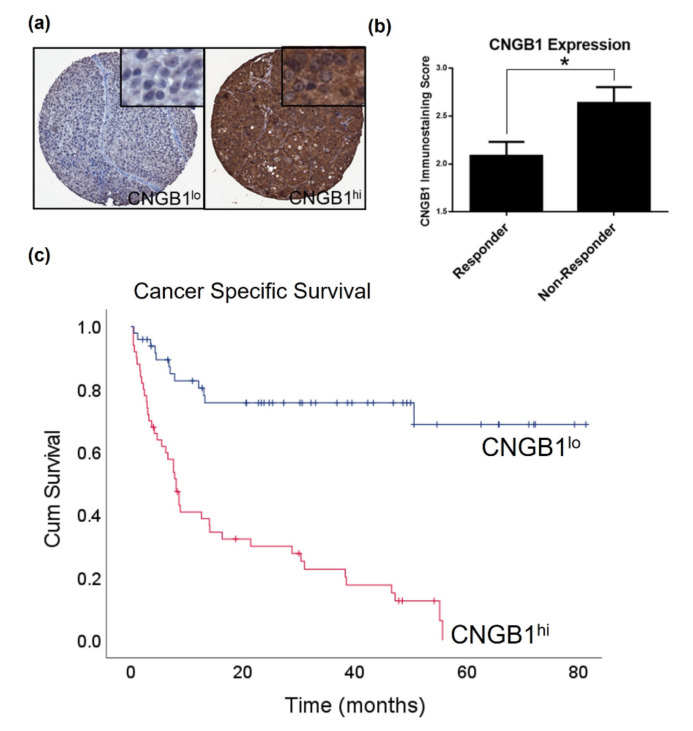
CNGB1 is upregulated in ‘Non-Responders’ to NAC and associates with MIBC patient survival. (**a**) Examples of low (CNGB1^lo^) and high (CNGB1^hi^) levels of CNGB1 expression in MIBC patient tissue cores. (**b**) Comparison of CNGB1 immunostaining score between ‘Responder’ and ‘Non-Responder’ patients, a protein-based validation of the Discovery cohort (*n* = 30, *t*-test, * *p* < 0.05). (**c**) Correlation of CNGB1 expression with cancer specific survival by Kaplan-Meier analysis (Validation cohort 2; *n* = 99, Log rank test, *p* < 0.001).

Since both machine learning and differential gene expression analysis suggest *CNGB1* as a marker of non-response to NAC, we next validated its expression using a tissue microarray comprising tumours from the 30 MIBC patients treated with NAC used in the gene expression profiling analysis (Discovery cohort); thus, acting as an internal validation on a protein level of the Discovery cohort (Figure 4a). Significant upregulation of CNGB1 immunostaining score was seen in ‘non-responders’ compared to ‘responders’ (‘responder’ score 2.1 ± 0.1 vs. ‘non-responder’ score 2.6 ± 0.2) (Figure 4b, *t*-test, *p* = 0.02). We further evaluated CNGB1 expression using a tissue microarray comprising tumours from our own cohort of 99 patients with MIBC (Validation cohort 2, Table S3). Kaplan-Meier survival analysis demonstrated that a high expression of CNGB1 (CNGB1^hi^, *n* = 50) associated with shorter CSS compared to low expressing patients (CNGB1^lo^, *n* = 49), indicating that high levels of CNGB1 expression were associated with a poor prognosis (Log rank test, *p* < 0.001, Figure 4c).

### 2.4. In Vitro Functional Validation of CNGB1 in MIBC Cells

**Figure 5:**
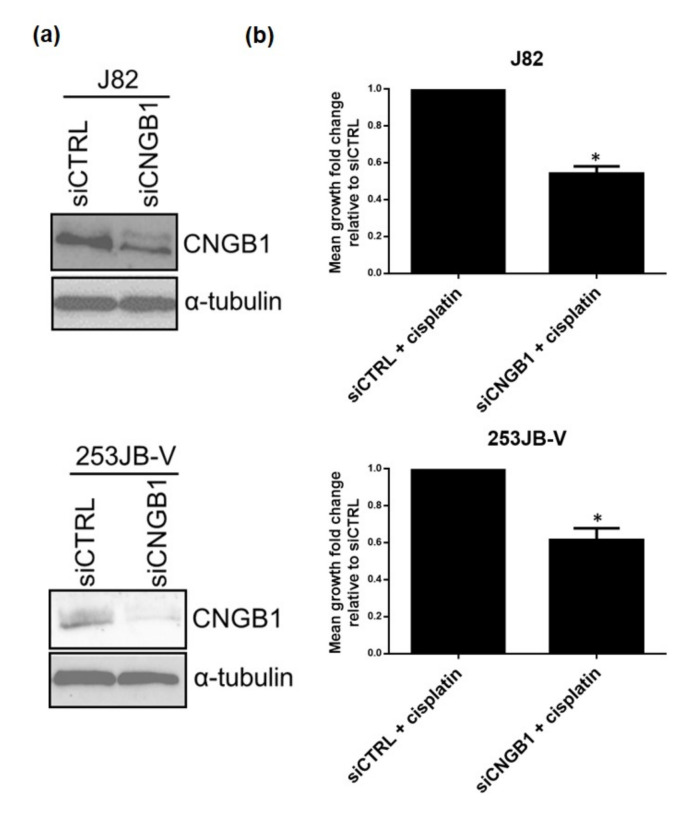
CNGB1 knockdown enhanced cisplatin sensitivity. (**a**) Confirmation of CNGB1 knockdown in J82 and 253JB-V MIBC cells by western blot analysis. α-tubulin was used as a loading control. (**b**) The effect of cisplatin, as a proxy for NAC, was assessed on the growth of J82 and 253JB-V cells following knockdown of CNGB1 for 48 h. Mean growth fold change relative to siCTRL was calculated for cisplatin treated cells (*n* = 3 experimental repeats, *t*-test, * *p* < 0.05).

Our two independent approaches of machine learning and differential gene expression analysis along with survival analysis of MIBC patient cohorts suggest *CNGB1* as a novel marker to predict response to NAC in MIBC patients. To determine whether *CNGB1* had a functional link to chemosensitivity in MIBC, in vitro validation studies were undertaken in MIBC cell lines, J82 and 253JB-V. Specifically, the effect of cisplatin, as a proxy for cisplatin-based NAC, (GI50 = 1.5 μM for J82 and GI50 = 2.5 μM for 253JB-V) on MIBC cell growth was assessed following knockdown of CNGB1 (siCNGB1) expression (Figure 5a, Figure S1). Knockdown of CNGB1 decreased growth of cisplatin treated MIBC cells compared to non-silencing control (siCTRL) cisplatin treated cells, -suggesting a role in cisplatin sensitivity (Figure 5b).

## 3. Discussion

To date, there are no clinically approved biomarkers predictive of response to NAC and identification of such predictors remains crucial for the selection of the most effective treatment for MIBC patients. To address this clinical urgency, a mutli-methods analysis approach of differential gene expression and machine learning methods was undertaken on a cohort of MIBC patients highly selected for an exquisitely strong chemotherapeutic response or marked resistance and/or progression. This approach identified a 9-gene signature able to select responders from non-responders with 100% accuracy which further showed significant association with survival in our limited external validation and also highlighted *CNGB1* as a promising potential biomarker to predict chemoresponsiveness of MIBC patients through validation in internal and external patient cohorts as well as in vitro studies.

Multiple signatures predicting response to NAC have been reported previously in MIBC, identifying markers such as *survivin*, *IPO7*, *TOP2A*, *PIR51*, *RACGAP1* and solute carriers such as *SLC16A3* and *SLC22A18* [13,15,16,17,18]. Even though none have been incorporated into routine clinical practice so far, they have provided positive steps towards achieving a precision medicine approach for the treatment of this disease. The signature identified in the present study appears mutually exclusive of previous studies, possibly reflecting the study design performed with the purposely selected patient cohort based on strict criteria for chemotherapeutic response. The interplay between bladder tumour biology, chemotherapy response and resistance mechanisms are complex. Recently, it has been suggested molecular subtyping may impact patient benefit to NAC [20,21,22,23]. Molecular subtypes have been discovered that are associated with specific clinicopathological characteristics and differential sensitivity to treatments. It will be interesting to interrogate expression of our signature and *CNGB1* in these subtypes particularly those expected to be resistant to NAC, such as the p53-like expression subtype.

Our analysis led to the identification of *CNGB1*, which encodes for one of the subunits that compose cyclic nucleotide-gated channels (CNGs). CNGs belong to the superfamily of voltage-gated ion channels and are key components for signal transduction by controlling the influx of cations, including Ca^2+^ ions, in response to signal-induced changes of cGMP or cAMP levels [31]. CNGs were first identified in retinal photoreceptors and olfactory sensory neurons, in which their function has been extensively studied [32,33]. Interestingly, Olfactory receptor 5P3 (*OR5P3*), a G-protein coupled-receptor also expressed by olfactory receptor neurons, was similarly upregulated in ‘non-responders’ and also identified in Signature 2 by RGIFE. CNGs expression has also been seen in other tissues including brain, liver and kidney, though their function in non-sensory cells is not as well understood [34]. Recently, a clinically aggressive variant of bladder cancer, sarcomatoid carcinoma, was shown to carry frequent mutations of *CNGB1* [35]. CNGs form heterotetramers composed of up to three different types of subunits that determine the channel’s functional features, including *CNGA1* which was also observed to be upregulated by ‘non-responders’ [36]. For example, rod photoreceptors comprise three CNGA1 subunit and one CNGB1 subunit, with the latter conferring Ca^2+^/Calmodulin-dependent modulation of channel activity. Upregulation of calcium-sensing proteins *CALML3*, *CALML5* and *S100A7A* was also noted in ‘non-responders’. Previously, a correlation of S100A7A (also known as psoriasin) expression with poor bladder cancer survival was seen [37]. A role for other S100 family of calcium-binding proteins in bladder cancer cisplatin sensitivity has also been reported [38,39]. Interestingly, *HIST1H4F*, identified by both RGIFE and differential gene expression analysis, has been shown previously to be part of a prognostic signature [40].

Calcium ions are one of the most important cellular messengers in biology and have been implicated many hallmarks of cancer [41]. Several drugs have been reported to block CNG channels including calcium channel blockers currently used in clinical practice in the management of high blood pressure, angina and cardiac arrhythmias, including dihydropyridines (e.g., nifedipine), phenylalkylamines (e.g., verapamil) and benzothiazepines (e.g., diltiazem) as well as the local anaesthetic tetracaine and calmodulin antagonists [32,42,43]. Calcium channel blockers can also enhance chemotherapy cytotoxicity by blocking the multidrug resistance protein P-glycoprotein, which through its function as an adenosine triphosphate-dependent drug efflux pump reduces intracellular chemotherapeutic drug accumulation [44]. Based on our proof-of-principle, future studies and clinical trials could explore calcium channel blockers as a clinical target for MIBC patients. Repurposing of approved calcium channel blockers could be a promising therapeutic approach for ‘non-responder’ MIBC patients to NAC.

Our multi-methods analysis approach of machine learning and differential gene expression analysis produced results consistent to each other and identified a signature and a candidate gene that appear to be promising and have provided an early compelling signal. However, we note that our validation focused primarily on *CNGB1* and investigation of our 9 gene signature (Signature 1) in other publicly available NAC-treated MIBC patient cohorts was limited. Additionally, determining whether the prediction accuracy of the signature is superior to that of the genes identified by differential gene expression analysis is warranted. Indeed, larger scale validation analysis is essential and would allow more definitive confirmation for the potential of the signature and *CNGB1* as a biomarker to predict chemoresponsiveness of MIBC patients.

## 4. Materials and Methods

### 4.1. Patient Cohorts

All patient tissue samples were used in accordance with approval granted by the Northumberland, Tyne and Wear NHS Strategic Health Authority Research Ethics Committee (reference 2003/11; The Freeman Hospital) and informed consent from all patients.

This study consisted of a MIBC patient sample set used for microarray gene expression profiling that was analysed by machine learning and differential gene expression approaches and further used for tissue microarray analysis (TMA, Discovery cohort). The demographic and clinicopathological characteristics are described in Table 1. All patients were histologically confirmed having pT2-T4G2-G3 transitional cell carcinoma of the bladder and were treated in the period from August 2002 to July 2010. A total of 30 samples were selected for microarray analysis (5 women and 25 men; median age, 64; range, 46–74 years). Patients underwent transurethral resection of bladder tumour and were referred for conventional 4 cycles of neoadjuvant platinum-based chemotherapy (MVAC). Patients underwent radical cystectomy and bilateral pelvic lymph node dissection. 

A published microarray dataset of MIBC patients was used for validation (GSE13507, Validation cohort 1 [30]).

An additional MIBC patient sample set (Validation cohort 2) was used also for validation by TMA analysis. The demographic and clinicopathological characteristics for this cohort, comprising 99 MIBC samples, are described in Table S3.

### 4.2. Tissue Sampling and Gene Expression Profiling

FFPE tissue samples were histologically examined to identify tumour regions. Sample cores (*n* ≥ 3) taken using a 0.6 mm core punch were deparaffinised in xylene for 5 min, rehydrated in 100% ethanol for 2 min (twice) and dried for 10 min at 55 °C. Tissue pellets were digested in 100 μL tissue lysis buffer with 16 μL 10% SDS and 40 μL Proteinase K overnight at 55 °C. Total RNA was extracted using High Pure RNA Paraffin kit (Roche, Basel, Switzerland). Gene expression profiling of samples was performed using Illumina’s Human Whole-Genome DASL HT Assay (Illumina, San Diego, CA, USA). Preparation of cDNA, DASL assay, hybridisation to Human HT-12 v4 expression beadchip, data collection and analysis (VisualSense) was carried out by Cambridge Genomic Services (GSC, Cambridge, UK).

### 4.3. Machine Learning Identification of Reduced Biomarker Panels

In order to identify candidate genes with good discriminative power between sample groups, we have used our own machine learning algorithm called RGIFE (Rank-Guided Iterative Feature Elimination). This algorithm was designed to identify reduced and highly discriminative panels of biomarkers from high-throughput omics data [27] and has shown good performance across a variety of scenarios including omics technologies (transcriptomics, proteomics) and diseases (cancer, osteoarthritis) [27,28,29]. RGIFE starts by considering all potential biomarkers as candidates, and iteratively some are dropped if their removal has no negative effect on the predictive capacity of a Random Forest (RF) machine learning algorithm. The predictive performance of the RF models is estimated from our data using a variant of stratified 10-fold cross-validation called DB-SCV (Distributed-balanced stratified cross-validation) [45] which is designed to create folds with more uniform data distribution than the standard cross-validation procedure, leading to more reliable estimates of predictive capacity as shown in [46]. This data partitioning process and predictive capacity estimation is performed within the RGIFE algorithm in order to select models having good predictive capacity on unseen data. The order in which features are removed is determined by the ranking of feature importance produced by RF as part of its training process. Initially RGIFE will attempt to remove blocks of 25% of the number of features. If the trial to remove a block fails (because its removal led to a model of worse quality) the algorithm will attempt to remove another block, following the ranking of features produced by RF. After five consecutive failed trials, the block size will be divided by 4 and the process will start again by attempting to remove the block at the bottom of the ranking. Once the block size is reduced to 1, the algorithm will stop after five unsuccessful trials and will return the remaining features as the final panel, hence automatically deciding when to stop the iterative feature elimination process. As this is a stochastic algorithm (due to both RF and the internal cross-validation process) running RGIFE multiple times can generate different biomarker panels. We have repeated this process five times to generate different biomarker signatures. For a full description of the algorithm see [27].

Furthermore, permutation tests were run to further evaluate the discriminative power of our signature of interest (Signature 1). To this aim we generated 1000 versions of the dataset in which the class labels (responder vs. non-responder) were scrambled. The purpose of this statistical test was to determine if this panel would be able to predict any set of class labels with the same distribution of responders and non-responders, i.e., if we break the link between inputs and outputs, can we still learn equally good models? For each permutation we trained and tested random forest models again using ten-fold cross-validation. After all permutations were generated and models trained and tested from these, the final step was to run a normality test to determine if the accuracy of 1 obtained from the original dataset would be part of the distribution of accuracies from the permutations. The permutations had an average accuracy was 0.58 ± 0.08, and the statistical tests was rejected with a *p*-value of 2.15 × 10^−12^.

SHAP (SHapley Additive exPlanations) is a game theoretic approach to explain the output of any machine learning model [47]. Using game theory, it fits a surrogate linear model on top of the RF model that enables the estimation of the contribution of each feature to each individual prediction made by the model, i.e., the SHAP values. Such values are estimated for each class in a dataset. If the SHAP value is positive for a class, it means that it is strongly contributing to the prediction of that class. A negative SHAP value reflects a lack of support for predicting such class.

### 4.4. Differential Expression Analysis

We used the limma R package to identify differentially expressed genes with the Empirical Bayes (eBayes) method to compute moderated *t*-statistics tests, adjusted for multiple comparisons with the Benjamini and Hochberg correction.

### 4.5. Immunostaining of Tissue Microarrays

Immunohistochemistry was performed using tissue microarrays containing 0.6-mm cores of MIBC and control tissues including breast, kidney, placenta, ovary and liver as described [48]. Sections were stained with anti-CNGB1 (1:2000; Novus Biologicals, Littleton, CO, USA) and viewed using Aperio CS2 (Leica Biosystems, Wetzlar, Germany). Negative controls were prepared by incubating without the primary antibody. Immunostaining was reviewed and scored independently by two assessors that were blinded to the clinical data to give average scores of staining intensity of absent (0), weak (1), moderate (2) or strong (3). High (CNGB1^hi^) and low (CNGB1^lo^) levels of CNGB1 expression were compared. ‘High’ expression was any single score ≥ 2.5, or an average score of ≥1.5 and ‘low’ expression was any score ≤ 1.

### 4.6. Cell Culture

Human bladder cancer cells J82 were obtained from the American Type Culture Collection (ATCC, HTB-1, Mannasas, VA, USA) and cultured in RPMI 1640 (Sigma, St Louis, MO, USA) supplemented with 10% fetal bovine serum (FBS) and 1% L-glutamine. The highly metastatic human TCC cell line 253JB-V was generously provided by Prof CP Dinney [49] and cultured in MEMalpha media supplemented with 5% FBS. All cells were grown at 37 °C in the presence of 5% CO_2_.

### 4.7. Western Blotting

Cells were lysed and analysed by SDS-PAGE as previously described [44]. Antibodies used where: anti-CNGB1 1:1000 (Millipore, cat. no. MABN282, Burlington, MA, USA) and anti-α-tubulin 1:4000 (Sigma, cat. no. T9026, St Louis, MO, USA).

### 4.8. siRNA Transfections

Cells were reverse transfected with siRNA using RNAiMax (Invitrogen, Waltham, MA, USA) according to the manufacturer’s instructions at a final concentration of 25 nM. siRNA sequence used for Scrambled (siCTRL) was 5′-UUCUCCGAACGUGUCACGUdTdT-3′. siRNA sequence used for CNGB1 (siCNGB1) was purchased from Dharmacon (product code J-006160-06-0002, Lafayette, CO, USA).

### 4.9. Cell Growth Assay

Cells were seeded into 96-well plates at a density shown to give exponential growth and approximately three cell doublings throughout the exposure period, that is 1 × 10^3^ cells/well for J82 cells and 2.5 × 10^3^ cells/well for 253JB-V cells, in 100 μL tissue culture medium. Six wells were seeded per experimental arm. Cells were reverse transfected with 25 nM siRNA using RNAiMax according to the manufacturer’s protocol (Invitrogen, Waltham, MA, USA). Following 48 h knockdown, cells were treated with cisplatin (GI50 = 1.5 μM for J82 and GI50 = 2.5 μM for 253JB-V) for 24 h. Cell viability was assessed with Sulforhodamine B (SRB) assay. Briefly, 50 μL of 50% trichloroacetic acid was added to each well and plates were incubated at 4 °C for 1 h. Plates were rinsed with distilled H_2_O and 100 μL of 0.4% SRB in 1% acetic acid was added to each well. Plates were incubated for 30 min at room temperature, and then rinsed with 1% acetic acid. SRB was solubilized with 100 μL of 10 mM Tris buffer pH 10.2 for 20 min with shaking. Absorbance values were measured on a microplate reader at 570 nm.

### 4.10. Statistical Analysis

Patient survival was analysed using the Kaplan-Meier method using the R survival package (SPSS, Chicago, IL, USA). For immunostaining score analysis and cell growth studies, the two-tailed paired *t*-test was used to determine statistical significance at a level of *p* < 0.05.

## 5. Conclusions

Our approach of multi-methods analysis of machine learning and differential gene expression analysis, using a purposely selected patient cohort based on strict criteria for chemotherapeutic response proceeded with validation in internal and external patient cohorts as well as in vitro studies, identified a novel gene signature able to select responders from non-responders with high predictive accuracy and highlighted *CNGB1* as a simpler proxy as a promising potential biomarker to predict chemoresponsiveness of MIBC patients. Our signature gene set and the role of *CNGB1* as a simpler proxy warrants additional larger scale validation in similar established cohorts of patients stratified based on response to NAC and ideally in trials of biomarker directed therapy in improving survival for MIBC patients.

## Figures and Tables

**Table 1:** Clinicopathological characteristics of MIBC patients examined by gene expression profiling.

Sample	Gender	Age	Preoperative Stage	Preoperative Grade	TNM Stage After RC	Response
good1	Male	71	T2	G2/3	T0 N0	Responder
good2	Female	68	T2	G2/3	T0 N0	Responder
good3	Male	74	T2	G3	Tcis N0	Responder
good4	Male	67	T2	G2/3	T0 N0	Responder
good5	Female	69	T2	G3	T0 N0	Responder
good6	Male	56	T2	G2	Tis N0	Responder
good7	Male	46	T2	G3	T0 N0	Responder
good8	Male	64	T2	G2/3	T0 N1 *	Responder
good9	Male	63	T2	G3	T0 N0	Responder
good10	Male	70	T4	G3	T0 N0	Responder
good11	Male	69	T2	G3	T0 N0	Responder
good12	Male	63	T2	G2	T1 N0	Responder
good13	Male	58	T1/2	G3	Tcis N0	Responder
good14	Male	60	T2	G3	T0 N0	Responder
good15	Male	59	T2	G3	T0 N0	Responder
good16	Female	70	T2	G3	Tis N0	Responder
good17	Male	69	T2	G3	T0 N0	Responder
good18	Female	73	T2	G3	T0 N0	Responder
good19	Male	59	T2	G3	T0 N0	Responder
bad1	Male	58	T2	G3	T4a N1	Non-Responder
bad2	Male	71	T2	G3	T3a N0	Non-Responder
bad3	Male	74	T2	G3	T3 N0	Non-Responder
bad4	Male	61	T2	G3	T3b N2	Non-Responder
bad5	Female	63	T2	G2/3	T3a N2	Non-Responder
bad6	Male	65	T2	G3	T2b N0	Non-Responder
bad7	Male	60	T2	G3	T3b N0	Non-Responder
bad8	Male	64	T1/2	G3	T2b N0	Non-Responder
bad9	Male	74	T2	G2	T3a N0	Non-Responder
bad10	Male	58	T2	G3	T3a N0	Non-Responder
bad11	Male	50	T2	G2/3	T4a N2	Non-Responder

TNM, Tumour, Node, Metastasis; RC, Radical Cystectomy; Response, response to neoadjuvant MVAC chemotherapy treatment; Responder, patient who achieved downstaging (≤pT1) after treatment; Non-Responder, patient who did not achieve downstaging (≥pT2) after treatment; *, micrometastatic. The *p* values for gender (Fisher’s exact, *p* = 0.626), age (*t*-test, *p* = 0.672), stage (Fisher’s exact, *p* = 1.000) and grade (Fisher’s exact, *p* = 1.000) were non-significant.

**Table 2:** List of signatures identified by machine learning algorithm RGIFE with high prediction accuracy.

Signature	Gene Symbol	Gene Name	Prediction Accuracy
1	*CNGB1*	cyclic nucleotide gated channel beta 1	1.000 (30/30*)
	*GGH*	gamma-glutamyl hydrolase	
	*HIST1H4F*	histone cluster 1 H4, family member F	
	*IDO1*	indoleamine 2,3-dioxygenase 1	
	*KIF5A*	kinesin family member 5A	
	*MRPL4*	mitochondrial ribosomal protein L4	
	*NCDN*	neurochondrin	
	*PRRT3*	proline-rich transmembrane protein 3	
	*SLC35B3*	solute carrier family 35, member B3	
2	*CNGB1*	cyclic nucleotide gated channel beta 1	1.000 (30/30*)
	*HIST1H4F*	histone cluster 1 H4, family member F	
	*NPEPPS*	aminopeptidase puromycin sensitive	
	*NUFIP2*	nuclear fragile X mental retardation protein interacting protein 2	
	*OR5P3*	olfactory receptor family 5, subfamily P, member 3	
	*PRRT3*	proline-rich transmemembrane protein 3	
	*TRMT1*	tRNA methyltransferase 1	
3	*CNGB1*	cyclic nucleotide gated channel beta 1	0.967 (29/30*)
	*HIST1H4F*	histone cluster 1 H4, family member F	
	*NR5A1*	nuclear receptor subfamily 5, group A, member 1	
	*PRRT3*	proline-rich transmembrane protein 3	
	*SLC2A4RG*	solute carrier family 2 member 4 regulator	
4	*CNGB1*	cyclic nucleotide gated channel beta 1	0.967 (29/30*)
	*HIST1H4F*	histone cluster 1 H4, family member F	
	*PRRT3*	proline-rich transmembrane protein 3	
	*TRMT1*	tRNA methyltransferase 1	
5	*CNGB1*	cyclic nucleotide gated channel beta 1	0.933 (28/30*)
	*HIST1H4F*	histone cluster 1 H4, family member F	
	*NR5A1*	nuclear receptor subfamily 5, group A, member 1	
	*PRRT3*	proline-rich transmembrane protein 3	

* Number of correctly classified samples over the total size of the dataset.

## Data Availability

Correlation was performed with survival data from GSE13507 [30].

## Supplementary Materials

The following are available online at https://www.mdpi.com/article/10.3390/cancers13153903/s1, Figure S1: Densitometry analysis, Table S1: Full list of genes upregulated (>3-fold) in ‘Responders’, Table S2: Full list of genes upregulated (>3-fold) in ‘Non-Responders’, Table S3: Clinicopathological characteristics of MIBC patients of Validation cohort 2.
