# Supplementary material for: Identification of CNGB1 as a Predictor of Response to Neoadjuvant Chemotherapy in Muscle-Invasive Bladder Cancer
Source: Cancers (Basel). 2021 Aug 2;13(15):3903. doi: 10.3390/cancers13153903 (PMC8345622; doi:10.3390/cancers13153903)
Supplement: Supplementary file 1 [file cancers-13-03903-s001.zip › cancers-1328818-supplementary.pdf]

cancers

MDPI

Supplementary Materials: Identification of CNGB1 as a Predictor of Response to Neoadjuvant Chemotherapy in Muscle-Invasive Bladder Cancer

Anastasia C. Hepburn, Nicola Lazzarini, Rajan Veeratterapillay, Laura Wilson, Jaume Bacardit and Rakesh Heer

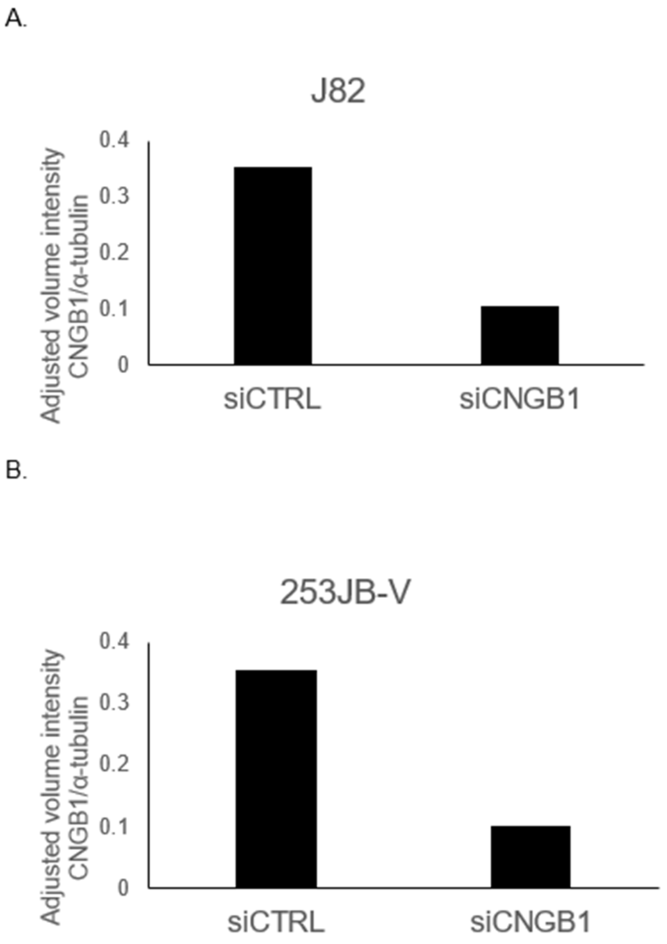

Figure S1. Densitometry analysis. Densitometry analysis performed using Image Lab software for western blots visualised with Biorad ChemiDoc.

Table S1. Full list of genes upregulated (>3-fold) in 'Responders'.

Rank	Probe ID	Gene Symbol	Gene Name	Fold Change
1	ILMN_1813490	*FSD1*	Fibronectin type III and SPRY domain containing 1	3.002926103
2	ILMN_2096031	*LOC283392*	Hypothetical protein	3.00371882
3	ILMN_2273053	*IL15*	Interleukin 15	3.046178387
4	ILMN_1746888	*PCOLCE2*	Procollagen C-endopeptidase enhancer 2	3.052867471
5	ILMN_1759652	*C1orf61*	Chromosome 1 open reading frame 61	3.11034367
6	ILMN_1703855	*SERPINB3*	Serpin family B member 3	3.12982915
7	ILMN_1737252	*NRG1*	Neuregulin 1	3.167735991
8	ILMN_1759622	*G3BP2*	G3BP stress granule assembly factor 2	3.178638308
9	ILMN_1722489	*TFF1*	Trefoil factor 1	3.208230197
10	ILMN_2119320	*HIST1H4F*	Histone cluster 1 H4 family member F	3.355192883

11	ILMN_1715169	*HLA-DRB1*	Major histocompatibility complex class II DR beta 1	3.687332254
12	ILMN_2165753	*HLA-A29.1*	Human leukocyte antigen (HLA)-A29	6.827009407
13	ILMN_2196479	*XRN2*	5'-3' Exoribonuclease 2	6.945393558

Table S2. Full list of genes upregulated (>3-fold) in 'Non-Responders'.

Rank	Probe ID	Gene Symbol	Gene Name	Fold Change
1	ILMN_3250412	*CNGB1*	Cyclic nucleotide gated channel beta 1	0.179950747
2	ILMN_2356578	*TH*	Tyrosine hydroxylase	0.211150055
3	ILMN_1804662	*NRG4*	Neuregulin 4	0.214592774
4	ILMN_1749118	*CALML5*	Calmodulin-like protein 5	0.225483892
5	ILMN_1677684	*BTBD16*	BTB/POZ domain-containing protein 16	0.237111786
6	ILMN_2140910	*OR5P3*	Olfactory receptor 5P3	0.247052675
7	ILMN_1660718	*GABBR2*	Gamma-aminobutyric acid type B receptor subunit 2	0.258141468
8	ILMN_1782141	*GRHL3*	Grainyhead-like 3	0.265609768
9	ILMN_1733333	*CALML3*	Calmodulin-like protein 3	0.27416442
10	ILMN_1815320	*NR5A1*	Nuclear receptor subfamily 5 group A member 1	0.275338343
11	ILMN_1717393	*PTCHD1*	Patched domain containing 1	0.277921703
12	ILMN_1687556	*DAPL1*	Death associated protein-like 1	0.283857875
13	ILMN_1669888	*CSTA*	Cystatin A	0.293359286
14	ILMN_1786353	*CNGA1*	Cyclic nucleotide-gated channel alpha 1	0.295413409
15	ILMN_1652366	*NLRP7*	NLR family pyrin domain containing protein 7	0.301105822
16	ILMN_1699809	*CAPNS2*	Calpain small subunit 2	0.303313065
17	ILMN_1793537	*MUC15*	Mucin 15	0.304246498
18	ILMN_3241282	*TDRD12*	Tudor domain containing 12	0.3073462
19	ILMN_1758888	*PADI3*	Peptidyl arginine deiminase, type III	0.308214192
20	ILMN_1717710	*SLC26A5*	Solute carrier anion transporter family 26 member 5	0.309348661
21	ILMN_1678186	*ATP6V0A4*	ATPase H+ Transporting V0 subunit A4	0.314885512
22	ILMN_2190541	*XAGE2B*	X antigen family, member 2B	0.31507576
23	ILMN_1701975	*SLC9A4*	Solute carrier family 9 member A4	0.317610722
24	ILMN_1680987	*HAND1*	Heart and neural crest derivatives expressed 1	0.32090989
25	ILMN_2133205	*GPX2*	Glutathione peroxidase 2	0.321564307
26	ILMN_1673191	*S100A7A*	S100 calcium-binding protein A7	0.321954615
27	ILMN_1759598	*DLX5*	Distal-less homeobox 5	0.323464337
28	ILMN_1742947	*SEC14L4*	SEC14p like lipid binding 4	0.325984882
29	ILMN_2246188	*SLC45A2*	Solute carrier family 45 member 2	0.328835443

Table S3. Clinicopathological characteristics of MIBC patients of Validation Cohort 2.

Characteristic	Patients (*n* = 99)
**Gender**	
Male	65 (65.7%)
Female	34 (34.3%)
**Age**	
Mean (range), years	73.8 (48-98)
**Clinicopathological stage at presentation**	
Localised	49 (49.5%)
Metastastic	50 (50.5%)

Western Blot Images

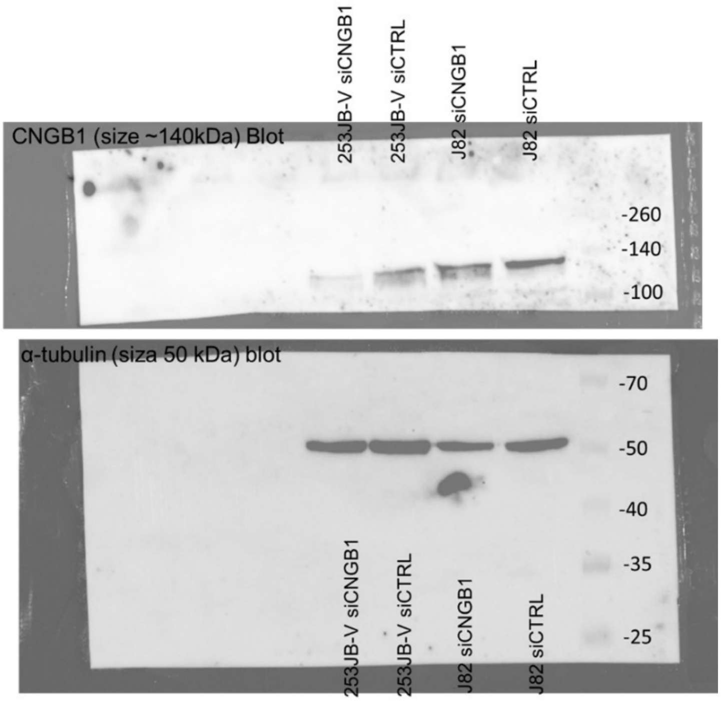

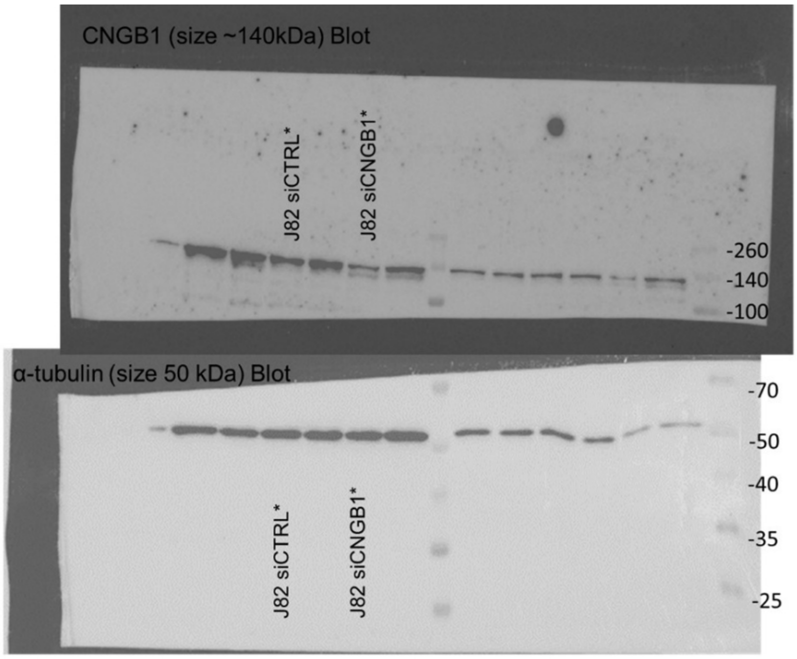

* Bands used in Figure 5. Repeat with more equal loading. Ladder used was Spectra™ Multicolor Broad Range Protein Ladder (Cat no 26623, ThermoScientific, Waltham, MA, USA).
